# Hydroxytyrosol-rich extract from olive juice as an additive in gilthead sea bream juveniles fed a high-fat diet: Regulation of somatic growth

**DOI:** 10.3389/fphys.2022.966175

**Published:** 2022-10-06

**Authors:** Sara Balbuena-Pecino, Manel Montblanch, Irene García-Meilán, Ramon Fontanillas, Ángeles Gallardo, Joaquim Gutiérrez, Isabel Navarro, Encarnación Capilla

**Affiliations:** ^1^ Departament de Biologia Cel·lular, Fisiologia i Immunologia, Facultat de Biologia, Universitat de Barcelona, Barcelona, Spain; ^2^ Skretting Aquaculture Research Centre, Stavanger, Norway

**Keywords:** olive polyphenols, aquafeeds, endocrine regulation, GH-IGF system, myogenesis, osteogenesis

## Abstract

The dietary inclusion of plant-based products in fish feeds formulation is required for the sustainable development of aquaculture. Moreover, considering functional diets, hydroxytyrosol, one of the major phenolic compounds found in olives (*Olea europaea*), has been identified as a potential candidate to be used in the aquafeeds industry due to its health promoting abilities. The aim of this study was to evaluate the effects of the inclusion of an olive juice extract rich in hydroxytyrosol as an additive (0.52 g HT/kg feed) in a high-fat (24% lipids) diet in gilthead sea bream (*Sparus aurata*) juveniles. Moreover, the experimental diets, with or without the extract, were administered daily at a standard (3% of total biomass in the tank) or restricted ration (40% reduction) for 8–9 weeks. Growth and biometric parameters, insulin-like growth factor 1 (IGF-1) plasma levels and growth hormone/IGF axis-, myogenic- and osteogenic-related genes expression in liver, white muscle and/or bone were analyzed. Moreover, *in vitro* cultures of vertebra bone-derived cells from fish fed the diets at a standard ration were performed at weeks 3 and 9 to explore the effects of hydroxytyrosol on osteoblasts development. Although neither body weight or any other biometric parameter were affected by diet composition after 4 or 8 weeks, the addition of the hydroxytyrosol-rich extract to the diet increased IGF-1 plasma levels, regardless of the ration regime, suggesting an anabolic condition. In muscle, the higher mRNA levels of the binding protein *igfbp-5b* and the myoblast fusion marker *dock5* in fish fed with the hydroxytyrosol-rich diet suggested that this compound may have a role in muscle, inducing development and a better muscular condition. Furthermore in bone, increased osteogenic potential while delayed matrix mineralization after addition to the diet of the olive juice extract was supported by the upregulated expression of *igf-1* and *bmp4* and reduced transcript levels of *osteopontin*. Overall, this study provides new insights into the beneficial use of hydroxytyrosol as a dietary additive in gilthead sea bream functional diets to improve muscle-skeletal condition and, the aquaculture industry.

## 1 Introduction

Olive (*Olea europaea*) oil is the major source of fats in the Mediterranean diet, and it is considered one of the main factors in the reduced occurrence of different chronic diseases, certain cancers, cardiovascular risk, and in the prolonged longevity associated to this diet (Sofi et al., 2010; Warleta et al., 2011; Marković et al., 2019). Its human health benefits are attributed to the presence of a high content of monounsaturated fatty acids (MUFAs), as well as many minor components that are functionally bioactive. These include tocopherols, phytosterols, carotenoids, triterpenic alcohols, pentacyclic triterpenes and phenolic compounds, which constitute the unsaponifiable fraction of the oil (Ghanbari et al., 2012). Nevertheless, in terms of health promoting abilities, polyphenols appear to be in the center of research interest (Marković et al., 2019). The main phenolic components in olive oil are hydroxytyrosol (3,4-dihydroxyphenylethanol), oleuropein and tyrosol (Tuck and Hayball, 2002) and, among them, hydroxytyrosol has shown the strongest antioxidant activity (Aydar et al., 2017). Besides, in mammals, hydroxytyrosol has been demonstrated to have anti-inflammatory (Gong et al., 2009), anti-obesogenic (Stefanon and Colitti, 2016; Illesca et al., 2019), antimicrobial (Medina et al., 2006) and anticancer (Corona et al., 2009; Fabiani et al., 2012) properties. In addition, olive polyphenols, especially hydroxytyrosol, exhibit a protective role in the prevention of osteoporosis, mainly due to stimulation of osteoblasts proliferation and differentiation while reducing the formation of osteoclasts (Chin and Ima-Nirwana, 2016; García-Martínez et al., 2016).

In the context of improving aquaculture sustainability, in addition to the shift toward the use of plant-derived ingredients in diets formulation instead of marine feedstuffs, the inclusion of phytocompounds in functional diets is nowadays uptrend. In this sense, olive oil inclusion in partially substituted fish oil diets has been evaluated in several fish species, including gilthead sea bream (*Sparus aurata*) (Torstensen et al., 2004; Mourente and Bell, 2006; Seno-o et al., 2008; Nasopoulou et al., 2011; Turchini et al., 2011). However, information about the potential benefits of using the extracts of olive pulp, oil or leaves or its chemical components as additives in aquafeeds is still scarce. Only recently, a triterpene-enriched olive extract in black sea bream (*Acanthopagrus schlegelii*) and an olive leaf extract in rainbow trout (*Oncorhynchus mykiss*) have shown immune potentiating properties (Baba et al., 2018; Rong et al., 2020). Furthermore, in gilthead sea bream, an olive oil bioactive extract added to the diet was demonstrated to improve gut health and functionality, as well as to enhance somatic growth (Gisbert et al., 2017).

In vertebrates, the main promoter of growth is the growth hormone (GH)-insulin-like growth factor (IGF) system (Reindl and Sheridan, 2012; Fuentes et al., 2013). The IGFs (IGF-1 and IGF-2) are produced in the liver in response to GH stimulation, are transported in circulation by the IGF binding proteins (IGFBPs), which modulate their half-life and actions, and exert their effects through the IGF-1 receptor (IGF-1R). Locally in skeletal muscle, hyperplastic growth is also controlled by myogenic regulatory factors (MRFs), with some being crucial for commitment of satellite cells [myogenic factor 5 (Myf5) and myogenic determination protein (MyoD)], while others contribute to myocytes differentiation [myogenic regulatory factor 4 (Mrf4) and myogenin] (Johnston, 2006; Vélez et al., 2014). Hydroxytyrosol and an olive leaf extract have been demonstrated, using *in vitro* and *in vivo* mammalian models, to have beneficial effects in muscle mass, thus protecting from sarcopenia (Salucci and Falcieri, 2020; González-Hedström et al., 2021). Indeed, some of these positive effects in rats consisted of reverting the aging-induced changes caused in expression of both, IGF system members (*igf-1r* and *igfbp5*) and myogenic markers (*myod* and *myogenin*). In fish, dietary supplementation with an olive leaf extract to red sea bream (*Pagrus major*) has been also reported to exert growth-promoting effects (i.e., hypertrophy) by increasing myofibril and collagen content in dorsal muscle (Arsyad et al., 2018).

Besides, coordinated growth between muscle and bone is required for proper musculoskeletal development. This has received increasing attention in farmed teleost species because skeletal anomalies are a major problem in the aquaculture industry (Witten et al., 2006). Runt-related transcription factor 2 (Runx2) is one of the key factors involved in the early stages of osteogenesis (Infante and Rodríguez, 2018). Afterwards, osteoblasts are known to produce different components of the extracellular matrix (ECM), including fibronectin (Fib1a) and collagen type 1 (Col1A1), and also non-collagenous proteins such as osteonectin (ON), osteopontin (OP) and osteocalcin (OCN), which are essential for osteoblasts maturation and matrix mineralization (Witten and Huysseune, 2009; Vieira et al., 2013). In fact, the bone protecting effects of olive oil phenolic compounds seem to be mediated by the modulation of osteoblasts markers expression (e.g., *runx2* and *ocn*) in mammalian cell models (Anter et al., 2016; Melguizo-Rodríguez et al., 2019). However, there is no information up to date about the specific effects of such polyphenols in the fish skeleton.

Furthermore, hydroxytyrosol has been demonstrated to have anti-obesogenic properties not only in mammals, but also in fish. In this sense, in a recent study by our group, hydroxytyrosol significantly decreased head and viscera adiposity of zebrafish larvae (*Danio rerio*), and it was able to counteract the obesogenic effects produced by rosiglitazone in primary cultured rainbow trout adipocytes (Lutfi et al., 2017). In terms of aquafeeds formulation, it is well known that within a certain range, increasing dietary lipids can promote fish development, spare dietary protein and reduce production costs (Zhang et al., 2017), whereas excessive dietary fat can cause metabolic alterations, fat deposition and inflammation, as reported in different fish species (Wang et al., 2015; Cao et al., 2019; Jin et al., 2020). Indeed, high-fat diets have been used as a model of obesity and related disorders in both mammals and fish (Hariri and Thibault, 2010; Seth et al., 2013; Salmerón et al., 2015; Ka et al., 2020). Specifically, in relation with the musculoskeletal system, long-term high-fat diet feeding can cause a decrease in swimming capacity and skeletal muscle fiber area, as well as muscular atrophy in zebrafish, generating a model of sarcopenic obesity (Zou et al., 2022). Besides, an imbalance of fat metabolism caused by a high-fat diet in the same species induced an increase in osteoclastic activity in the exoskeleton, generating an osteoporosis-like phenotype (Carnovali et al., 2018). Nevertheless, further investigation about the role of hydroxytyrosol modulating adiposity in fish is beyond the scope of the present manuscript, since this is part of a larger study. Therefore, since hydroxytyrosol has demonstrated multiple positive effects at different tissues, we considered when setting the trial, to evaluate fish fed a high-fat diet, and administered at two rations, standard (*ad libitum*) or restricted, to establish two different conditions in terms of energy intake and to test whether the effects of hydroxytyrosol on muscle and bone homeostasis may vary in the two nutritional situations. Thus, our hypothesis in this first study, considering the beneficial properties of hydroxytyrosol, is that the dietary administration of an extract rich in this polyphenol to gilthead sea bream could prevent the possible alterations associated to a high-fat diet feeding by improving the muscular and skeletal growth capacity of the fish.

In this framework, the objective of this study was to evaluate whether the inclusion of an olive juice extract rich in hydroxytyrosol in a high-lipid content diet could improve musculoskeletal growth of gilthead sea bream juveniles fed at standard or reduced ration. To this end, changes in growth and somatic parameters, IGF-1 plasma levels, as well as in the mRNA levels of members of the GH-IGF axis, and myogenic and osteogenic markers in muscle and/or bone were analyzed. Moreover, modulation of proliferation and differentiation of bone-derived cells from the same fish was also assessed to evaluate if the *in vivo* feeding history of the fish could affect the osteogenic development of bone-derived cells *in vitro*.

## 2 Materials and methods

### 2.1 Animals and experimental design

Gilthead sea bream (*Sparus aurata*) juveniles were obtained from the commercial fishery Piscimar (Burriana, Spain) and maintained in the animal facilities of the Faculty of Biology at the University of Barcelona (Spain). Animals were kept in a seawater recirculation system at 23 ± 1°C, under a 12 h light/12 h dark photoperiod, and fed *ad libitum* two times a day with a commercial diet (Optibream Skretting, Burgos, Spain). After 1 month of acclimation, fish were weighed and distributed between eight 200 L (*n* = 15 fish for each tank) and four 400 L (*n* = 30 fish per tank) tanks. Before starting the trial, fish were fed *ad libitum* with the experimental diet without the hydroxytyrosol-rich extract for 1 week, in order to have the fish habituated to it, but also, to identify the ration to which the visual satiation level corresponded to set it in the experimental trial as the standard one. The initial body weight (BW) and biomass density of fish were 80.81 ± 1.43 g and 6.06 kg/m^3^, respectively.

The experimental high-fat diet (24% lipids of the dry matter) was formulated and produced by Skretting ARC (Stavanger, Norway) with 50% of total oils being rapeseed oil, the other 50% fish oil, and 23 MJ/kg of digestible energy (Table 1). Moreover, this diet was formulated in the absence (HF) or presence (HF + HT) of a hydroxytyrosol-rich extract (0.52 g HT/kg feed), and was administered daily at a standard ration (3% of total biomass in the tank) (ST) or at restricted conditions (40% reduction) (RE) for 8–9 weeks (from the end of August to the end of October). Each of the four experimental groups of fish was distributed in triplicate tanks (one of 400 L and two of 200 L due to the particular tanks distribution at the fish facility). The olive juice extract rich in hydroxytyrosol used in the formulation (HIDROX^®^ > 12% simple and total polyphenols: 3.136% HT, 0.216% oleuropein and 0.408% tyrosol; according to the certificate of analysis 12-190403-000) was provided by Oliphenol LLC., (Hayward, CA, United States). The dose of 0.52 g HT/kg feed was selected according to data presented from an experiment performed in the yellowtail kingfish (*Seriola lalandi*) at an international conference (Bas et al., World Aquaculture Society Congress 2019). The groups that received the standard ration (HF_ST and HF + HT_ST) were fed two times a day, with 60% of the feed in the morning ration and 40% in the afternoon, whereas the ones with the reduced regime (HF_RE and HF + HT_RE) were fed only once a day in the morning with 60% of the total standard ration. Daily ration relative to the BW was adjusted every 2 weeks. The main trial lasted 8 weeks, but some fish from the groups HF_ST and HF + HT_ST were maintained for an extra week in order to perform the *in vitro* experiments. Those were performed on weeks 3 and 9 to avoid overlapping with the fish samplings.

**TABLE 1:** Composition of the experimental high-fat diets without (HF) or with hydroxytyrosol (HF + HT). The hydroxytyrosol included in the formulation (0.52 g HT/kg feed) was provided by the commercial olive juice extract HIDROX^®^ (Oliphenol LLC., Hayward, CA, United States) that contains a >12% of simple and total polyphenols from which 3.136% is hydroxytyrosol.

	HF	HF + HT
**Ingredients (%)**		
Corn gluten	3.80	3.80
Wheat gluten	20.00	20.00
Fava beans	8.00	8.00
Soya concentrate	25.00	25.00
Fish oil	9.98	9.98
Fish meal	15.00	15.00
Rapeseed oil	10.14	10.14
Yttrium premix	0.10	0.10
Phosphate	1.04	1.04
Vitamin mineral premix	0.44	0.44
Wheat	6.50	4.85
HIDROX^®^	0.00	1.66
**Composition (%)**		
Dry matter	93.0	93.0
Moisture	7.0	7.0
Crude protein	46.8	46.7
Crude fat	24.0	24.2
Ash	5.4	5.6
Crude fiber	1.9	1.8
Starch	8.8	7.8

At sampling, fish were fasted for 24 h in order to deplete the gastrointestinal tract and avoid contamination of the tissues. Ten animals per condition (four and three from the 400-L and 200-L tanks, respectively) were anesthetized with MS-222 (150 mg/L) (E10521, Sigma-Aldrich, Tres-Cantos, Spain), measured, weighed, and blood was taken from the caudal vessels. Samples of blood were immediately placed on ice and centrifuged at 5,000 rpm for 5 min at room temperature to obtain the plasma. After sacrifice by sectioning the spinal cord, samples of white skeletal muscle, vertebrae bone and liver were obtained and directly frozen in liquid nitrogen. All samples were stored at −80°C until further analysis. All animal handling procedures carried out in this study complied with the Guidelines of the European Union Council directive (EU 2010/63) and were approved by the Ethics and Animal Care Committee of the University of Barcelona (permit numbers CEEA 34/20 and DAAM 11251), following the regulations and procedures established by the Spanish and Catalan governments.

### 2.2 Biometric parameters

BW was monitored at times 0, 2, 4, 6 and 8 weeks. The rest of the biometric parameters were measured at the middle (4 weeks) and end (8 weeks) of the experimental trial. BW and standard length (BL) were used to determine the condition factor (CF) [BW/BL^3^ × 100] individually, although the average values for each tank (*n* = 3) were used for statistical analysis. Similarly, weight of the liver was obtained, and the hepatosomatic index (HSI) [(liver weight/BW) × 100] calculated and averaged per each tank (*n* = 3). Total biomass from triplicate tanks (*n* = 3) was obtained to calculate feed conversion ratio (FCR), as grams of total feed intake/(final BW- initial BW).

### 2.3 Insulin-like growth factor 1 plasma levels

IGF-1 plasma levels were determined in 10 fish per group at the middle (4 weeks) and end (8 weeks) of the experimental trial after sample extraction by the acid-ethanol method (Shimizu et al., 2000). The concentration of IGF-1 was measured using the Fish IGF-1 ELISA kit (CSB-E12122Fh, CUSABIO, Wuhan, China), according to the manufacturer’s instructions, as previously done with plasma samples of the same species by Rodríguez-Viera et al. (2022).

### 2.4 Gene expression analyses

#### 2.4.1 RNA extraction and cDNA synthesis

White skeletal muscle (∼100 mg) and vertebral bone tissues (∼100 mg) were homogenized with 1 ml of TRI Reagent Solution (Applied Biosystems, Alcobendas, Spain) using the Precellys^®^ Evolution Homogenizer coupled to a Cryolys cooling unit (Bertin Instruments, Montigny-le-Bretonneux, France). Next, RNA extraction was performed according to the manufacturer’s recommendations and resuspended in DEPC-treated water (RNase-free). RNA concentration of each sample was measured in a NanoDrop 2000 (Thermo Scientific, Alcobendas, Spain), and its integrity was confirmed in a 1% agarose gel (w/v) stained with 3% SYBR^®^-Safe Gel Stainer (Bio-Rad, El Prat de Llobregat, Spain). Afterwards, 1,500 ng of RNA were treated with DNase I (Life Technologies, Alcobendas, Spain), to remove any residual genomic DNA, and retrotranscribed with the Transcriptor First Strand cDNA Synthesis Kit (Roche, Sant Cugat del Vallès, Spain).

#### 2.4.2 Real-time quantitative polymerase chain reaction

Gene expression analyses were performed by quantitative polymerase chain reaction (qPCR) in a CFX384^TM^ Real-Time System (Bio-Rad, El Prat de Llobregat, Spain), according to the requirements of the MIQE guidelines (Bustin et al., 2009). The primers sequences and GenBank accession numbers used for each reaction are listed in Supplementary Table S1. All the analyses were performed in triplicate wells using 384-well plates with 2.5 μL of iTaq Universal SYBR Green Supermix (Bio-Rad, El Prat de Llobregat, Spain), 250 nM of forward and reverse primers and 1 μL of diluted cDNA for each sample, in a final volume of 5 μL. The qPCR program was performed as previously described (Balbuena-Pecino et al., 2019). The level of expression of each target gene was calculated relative to the geometric mean of the two most stable reference genes from the three determined for each tissue [ribosomal protein s18 (*rps18*) and elongation factor 1 alpha (*ef1α*) in all three tissues], according to the Pfaffl method (Pfaffl, 2001). Reference genes stability and relative expression of the target genes were determined using the Bio-Rad CFX Manager Software v. 3.1.

### 2.5 Primary culture of bone-derived cells

Primary cultures of gilthead sea bream bone-derived cells were performed following the protocol of Capilla et al. (2011). After 3 and 9 weeks of the experimental trial, vertebral columns of 8 fish per dietary condition (HF and HF + HT at standard ration) were used per culture, each one considered an independent replicate. After cleaning the vertebral column of all adherent tissues, small fragments (<1 mm) were obtained by mechanical disruption. After that, two digestions of 30 and 90 min were done with 0.125% type II collagenase in Hank’s balanced salt solution with gentle agitation. Next, bone fragments were washed twice with Dulbecco’s Modified Eagle Medium (DMEM) and plated in growth medium composed of DMEM supplemented with 10% fetal bovine serum and 1% antibiotic/antimycotic solution in 12-well plates, and incubated at 23°C with 2.5% CO_2_. The bone fragments were removed at day 7 of culture development in order to perform the corresponding assays with the attached cells. Medium was changed every 2 days. All plastic materials were obtained from Nunc (LabClinics, Barcelona, Spain).

### 2.6 Proliferation assay

The methylthiazolyldiphenyl-tetrazolium bromide (MTT) assay was used to assess cell proliferation, as previously done with the same cellular model and species (Balbuena-Pecino et al., 2019). Briefly, on days 8 and 15, bone-derived cell samples of two-duplicate wells from the 12-well plates were incubated for 3 h in DMEM with a final concentration of 0.5 mg/ml of MTT (M5655, Sigma-Aldrich, Tres-Cantos, Spain). Then, cells were washed with phosphate buffered saline and the blue formazan crystals formed were allowed to resuspend in dimethyl sulfoxide. The normalized proliferation values (*n* = 8) were obtained from the absorbance measured at 570 nm in duplicate 96-wells, subtracting the background read at 650 nm, using a microplate reader (Tecan Infinite M200, Männedorf, Switzerland).

### 2.7 Differentiation assay

Cell differentiation was evaluated according to mineralization of the ECM. The deposition of minerals was analyzed by alizarin red S (ARS) staining, as previously described in Capilla et al. (2011). Briefly, at day 20, cell samples of two-duplicate wells from 12-well plates were fixed with 10% formalin, rinsed three times with distilled water and stained with 2% ARS (pH 4.1-4.3) for 20 min. Next, cells were washed three times with distilled water, and a 10% acetic acid solution was used to elute the ARS dye with the help of a scrapper. Then, cells were heated at 85°C, cooled on ice and centrifuged at 16,000 g for 15 min. At this point, 10% ammonium hydroxide was added to the supernatant to neutralize the acid and aliquots of the different samples were read in triplicate 96-wells at 405 nm, using a microplate reader (Tecan Infinite M200, Männedorf, Switzerland). Data are presented as optical density arbitrary units (*n* = 8).

### 2.8 Statistical analyses

Data were analyzed using IBM SPSS Statistics v. 22 (IBM Corp., Armonk, NY, United States) and plotted as Mean + Standard Error of the Mean (SEM) using GraphPad Prism v. 7 (GraphPad Software, La Jolla, CA, United States). As a biological replicate, tank was used for biometric parameters and individual fish for IGF-1 plasma levels, gene expression and *in vitro* results. Data normality and homoscedasticity were checked with Shapiro-Wilk and Levene’s tests, respectively. Next, statistical differences were assessed by two-way analysis of variance (two-way ANOVA) with diet and ration set as independent factors, and their interaction. When the interaction between the two factors was significant, comparisons among groups were analyzed by a Tukey’s *post-hoc* test. With regards to *in vitro* assays, statistical significance between dietary groups was performed with unpaired student t-tests. Statistical differences were considered significant for all analyses when *p* < 0.05.

## 3 Results

### 3.1 Growth and somatic parameters

**FIGURE 1:**
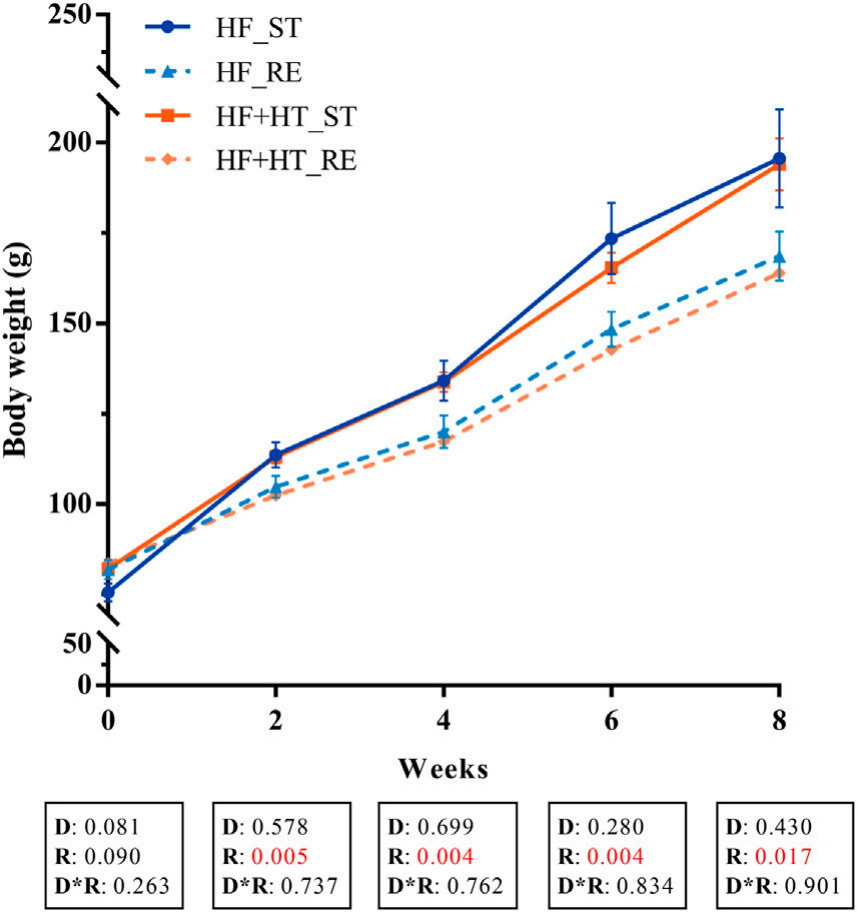
Mean body weight of gilthead sea bream juveniles fed with a high-fat diet in the absence (HF) or presence (HF + HT) of hydroxytyrosol (0.52 g HT/kg feed), at a standard (ST) (3% biomass/tank) or restricted ration (RE) (40% daily reduction), for 0, 2, 4, 6 and 8 weeks. Values are presented as Mean ± SEM (*n* = 3 tanks). Statistical differences are indicated in 3 components: diet (D), ration (R) and interaction (D*R), using two-way ANOVA (*p* < 0.05, showed in red).

BW was measured every 2 weeks along the experimental trial and values are presented in Figure 1. Two-way ANOVA analysis showed that BW was significantly affected by ration regime but not by the diet formulation from week 2 to the end of the experiment. During this period, fish fed with the standard ration (HF_ST and HF + HT_ST) presented higher BW values compared to those fish fed with the restricted ration (HF_RE and HF + HT_RE) (Figure 1). Besides BW, other somatic indices together with the IGF-1 plasma levels, evaluated at weeks 4 and 8, are shown in Tables 2, 3, respectively. At week 4, under restricted feeding, fish had significantly lower values of CF, FCR, liver weight and HSI regardless of the diet. Moreover, the circulating IGF-1 plasma levels were affected by the diet composition, and were significantly higher in fish fed with the diet containing the hydroxytyrosol-rich extract in comparison to fish fed with HF diet at both feeding regimes. On the other hand, BL was not affected by any factor (Table 2).

**TABLE 2:** Somatic indices and IGF-1 plasma levels of gilthead sea bream juveniles fed with a high-fat diet in the absence (HF) or presence (HF + HT) of hydroxytyrosol (0.52 g HT/kg feed), at a standard (ST) (3% biomass/tank) or restricted ration (RE) (40% daily reduction) for 4 weeks.

	HF_ST	HF_RE	HF + HT_ST	HF + HT_RE	D	R	D*R
BW (g)	134.14 ± 5.54	120.04 ± 4.47	133.81 ± 2.78	117.30 ± 0.52	0.699	0.004	0.762
BL (cm)	16.96 ± 0.14	16.59 ± 0.27	17.19 ± 0.13	16.68 ± 0.21	0.444	0.057	0.730
CF	2.85 ± 0.05	2.57 ± 0.04	2.80 ± 0.09	2.64 ± 0.05	0.266	<0.001	0.569
FCR	1.53 ± 0.34	0.97 ± 0.09	1.53 ± 0.11	1.05 ± 0.04	0.841	0.024	0.827
Liver (g)	1.91 ± 0.11	1.43 ± 0.09	2.03 ± 0.17	1.51 ± 0.04	0.393	0.002	0.868
HSI (%)	1.39 ± 0.06	1.21 ± 0.08	1.34 ± 0.03	1.25 ± 0.05	0.976	0.049	0.442
IGF-1 (ng/ml)	3.27 ± 0.16	3.19 ± 0.18	3.88 ± 0.09	3.72 ± 0.10	<0.001	0.417	0.773

Data are shown as Mean ± SEM (*n* = 3 tanks, except for IGF-1 plasma levels *n* = 10 fish). Statistical differences are indicated in 3 components: diet (D), ration (R) and interaction (D*R), using two-way ANOVA (*p* < 0.05). BW, body weight; BL, body length; CF, condition factor (BW/BL^3^) × 100; FCR, feed conversion ratio (g total feed intake/(BW- initial BW); HSI, hepatosomatic index (liver weight/BW) × 100.

**TABLE 3:** Somatic indices and IGF-1 plasma levels of gilthead sea bream juveniles fed with a high-fat diet in the absence (HF) or presence (HF + HT) of hydroxytyrosol (0.52 g HT/kg feed), at a standard (ST) (3% biomass/tank) or restricted ration (RE) (40% daily reduction) for 8 weeks.

	HF_ST	HF_RE	HF + HT_ST	HF + HT_RE	D	R	D*R
FBW (g)	195.63 ± 13.54	168.65 ± 6.81	194.00 ± 7.24	163.96 ± 1.40	0.430	0.017	0.901
BL (cm)	19.04 ± 0.40	18.34 ± 0.09	19.31 ± 0.17	18.09 ± 0.34	0.977	0.009	0.381
CF	2.85 ± 0.04	2.75 ± 0.06	2.78 ± 0.05	2.66 ± 0.06	0.203	0.085	0.794
FCR	1.66 ± 0.25	0.99 ± 0.06	1.68 ± 0.15	1.08 ± 0.10	0.744	0.004	0.822
Liver (g)	2.42 ± 0.14	2.08 ± 0.20	2.56 ± 0.21	1.96 ± 0.20	0.973	0.036	0.523
HSI (%)	1.23 ± 0.05	1.20 ± 0.09	1.29 ± 0.003	1.23 ± 0.06	0.466	0.475	0,859
IGF-1 (ng/ml)	3.88 ± 0.13^a^	4.70 ± 0.12^b,c^	4.97 ± 0.10^c^	4.44 ± 0.11^b^	0.001	0.225	<0.001

Data are shown as Mean ± SEM (*n* = 3 tanks, except for IGF-1 plasma levels *n* = 10 fish). Statistical differences are indicated in 3 components: diet (D), ration (R) and interaction (D*R), using two-way ANOVA (*p* < 0.05). When the interaction between the two factors (D*R) was significant, comparisons among groups were analyzed by a Tukey’s post-hoc test and, significant differences are indicated by different letters (*p* < 0.05). FBW, final body weight; BL, body length; CF, condition factor (FBW/BL^3^) × 100; FCR, feed conversion ratio (g total feed intake/(FBW- initial BW); HSI, hepatosomatic index (liver weight/FBW) × 100.

At the end of the experimental trial (week 8), the ration factor was found to significantly affect BL, FCR and liver weight. Both groups of fish fed with a restricted regime had lower values in comparison with their respective fish groups fed at a standard ration. Concerning IGF-1, a diet effect and an interaction between the two factors was observed. In this sense, the highest value of circulating IGF-1 was found in the group fed the HF + HT diet and the lowest in the HF-fed fish when both groups received a standard ration. Animals fed with the HF + HT diet presented higher values of circulating IGF-1 when fed the standard ration compared to the restricted one, but the situation was the opposite in the animals fed with the HF diet. Finally, CF and HSI parameters were unaffected either by diet or ration (Table 3).

### 3.2 Gene expression related to GH-IGFs axis

**FIGURE 2:**
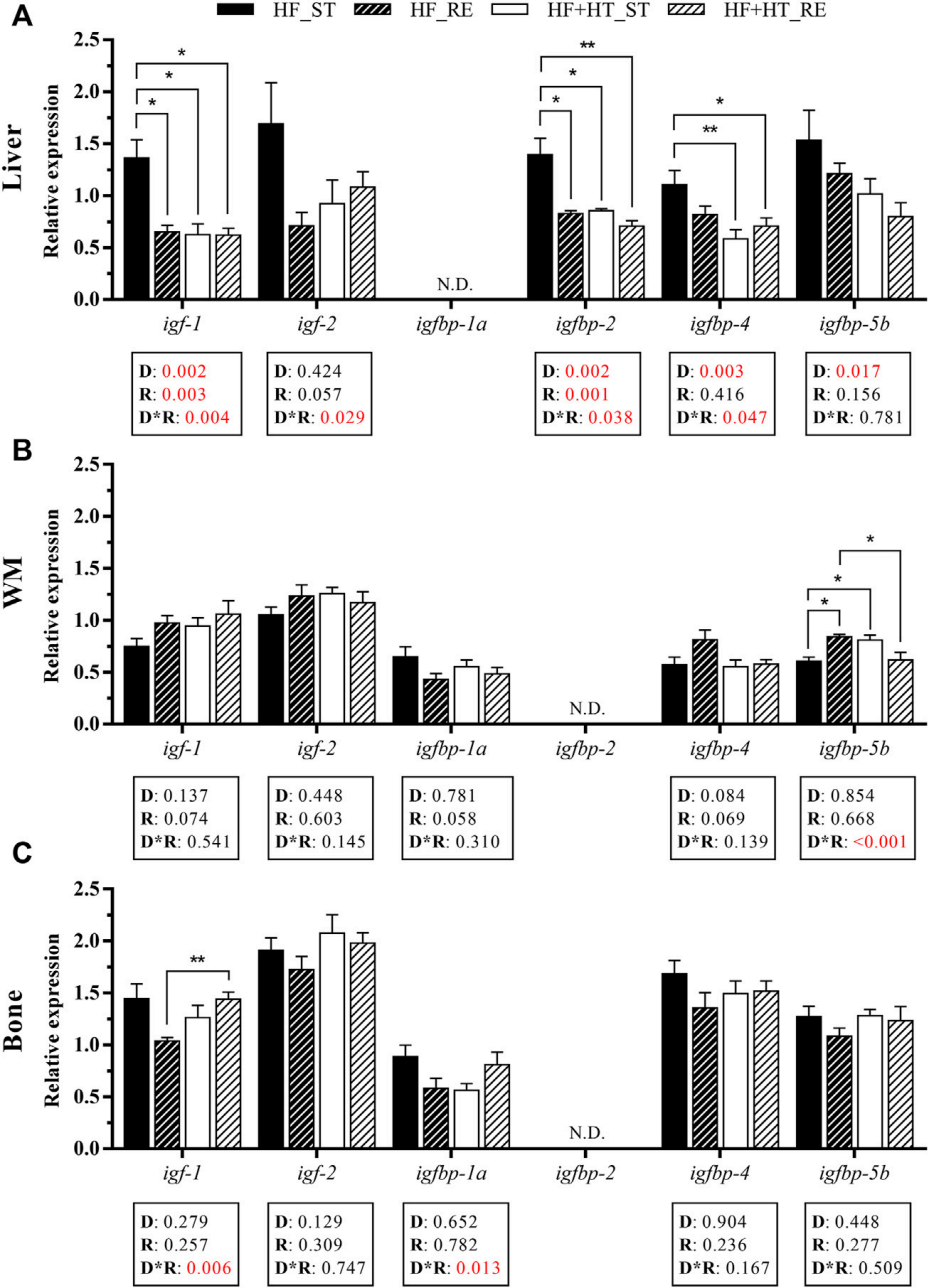
Relative gene expression of insulin-like growth factors (IGFs) and IGF binding proteins (IGFBPs) in **(A)** liver, **(B)** white muscle (WM) and **(C)** bone of gilthead sea bream juveniles fed with a high-fat diet in the absence (HF) or presence (HF + HT) of hydroxytyrosol (0.52 g HT/kg feed), at a standard (ST) (3% biomass/tank) or restricted ration (RE) (40% daily reduction) for 8 weeks. Data are shown as Mean + SEM (*n* = 10). Statistical differences are indicated in 3 components: diet (D), ration (R) and interaction (D*R), using two-way ANOVA (*p* < 0.05, showed in red). When the interaction between the two factors (D*R) was significant, comparisons among groups were analyzed by a Tukey’s post-hoc test and, significant differences are indicated by asterisks (*p* < 0.05 shown as *; *p* < 0.01 **). N.D., non-detectable.

In the liver, the transcript levels of *igf-1* and the IGF binding protein *igfbp-2* were affected by the diet composition, the feeding ration, and by the interaction between both factors. Hence, multiple comparisons analysis revealed that the expression of these two genes was significantly higher in HF_ST fish respect to the other three groups. Moreover, a diet effect was also found in *igfbp-4* and *igfbp-5b*, presenting both genes higher expression levels in the animals fed the HF diet compared to those fed with the HF + HT diet regardless of the feeding regime. In addition, *igf-2* and *igfbp-4* showed interaction of both variables. Particularly, fish fed with the HF diet at the standard ration had significantly increased mRNA levels of *igfbp-4* when compared with both groups of HF + HT diet-fed fish. Finally, *igfbp-1a* levels could not be detected on this tissue (Figure 2A).

With regards to white muscle, a significant interaction between diet and ration regime was observed in the mRNA levels of *igfbp-5b*, which were significantly higher in the HF_RE and HF + HT_ST groups compared to fish fed with the HF diet at standard ration, and also, with HF + HT_RE group in the case of the HF_RE group. Apart from this, differences were not found in the case of *igf-1*, *igf-2*, *igfbp-1a* and *igfbp-4*, whereas *igfbp-2* was undetectable in this tissue (Figure 2B).

In bone, there was a significant interaction between diet and feeding ration in the gene expression of *igf-1* and *igfbp-1a*; thus, transcript levels of the former were higher in the HF + HT_RE group compared to HF_RE fed fish. However, the mRNA levels of *igf-2*, *igfbp-4* and *igfbp-5b* were not affected by any of the factors, and *igfbp-2* gene expression was again undetectable (Figure 2C).

**FIGURE 3:**
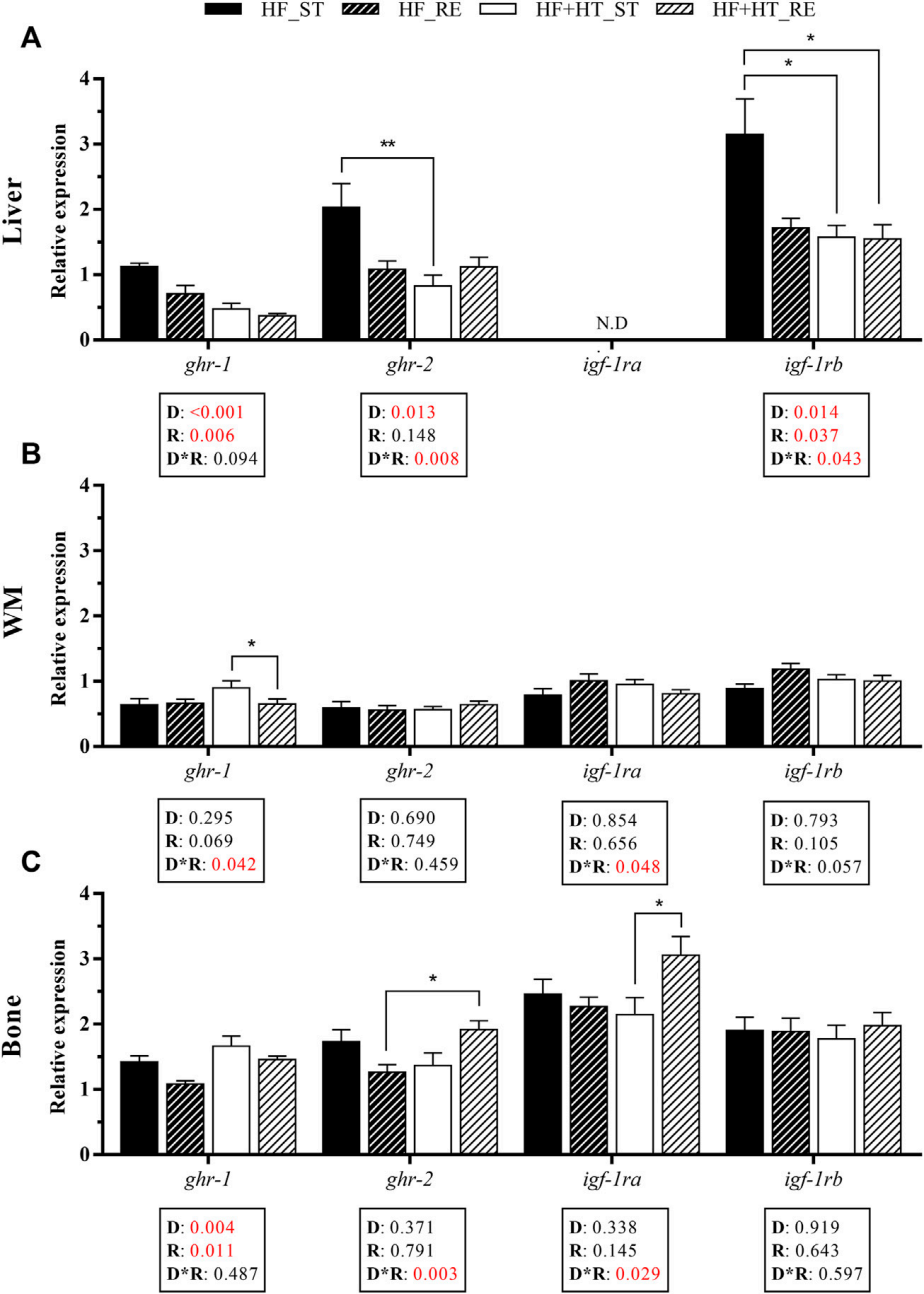
Relative gene expression of growth hormone (GH) and insulin-like growth factor (IGF-1) receptors in **(A)** liver, **(B)** white muscle (WM) and **(C)** bone of gilthead sea bream juveniles fed with a high-fat diet in the absence (HF) or presence (HF + HT) of hydroxytyrosol (0.52 g HT/kg feed), at a standard (ST) (3% biomass/tank) or restricted ration (RE) (40% daily reduction) for 8 weeks. Data are shown as Mean + SEM (*n* = 10). Statistical differences are indicated in 3 components: diet (D), ration (R) and interaction (D*R), using two-way ANOVA (*p* < 0.05, showed in red). When the interaction between the two factors (D*R) was significant, comparisons among groups were analyzed by a Tukey’s post-hoc test and, significant differences are indicated by asterisks (*p* < 0.05 shown as *; *p* < 0.01 **). N.D., non-detectable.

With respect to the transcriptional profile of the receptors from the GH-IGF axis in liver, the mRNA levels of the GH receptor *ghr-1* were modulated by the diet composition and the feeding ration. An upregulation of this gene was observed in the HF group compared to the HF + HT one, with these levels being reduced by restriction regime (HF_RE and HF + HT_RE). Similarly, a diet effect and an interaction between the two factors was found for *ghr-2* gene expression. In this sense, an upregulation of *ghr-2* mRNA levels was detected in animals fed with HF diet at a standard ration compared to those in fish fed with HF + HT diet at the same ration. Moreover, the transcript levels of the IGF-1 receptor *igf-1rb* were altered by the diet composition, the feeding regime and, by the interaction of both factors. *igf-1rb* mRNA levels were higher in fish that received the HF diet at a standard ration in comparison with both groups of HF + HT diet. On the other hand, *igf-1ra* levels could not be detected on this tissue (Figure 3A).

In the case of white muscle, the interaction between diet and feeding ration affected *ghr-1* and *igf-1ra* gene expression and, in the case of *ghr-1*, higher levels were found in HF + HT-fed fish at a standard ration compared with those in fish fed with the same diet but at the restricted regime. The mRNA levels of the other receptors were not modified (Figure 3B).

In bone, diet composition and feeding ration had significant effects on *ghr-1*, and an interaction effect was observed in *ghr-2* and *igf-1ra* gene expression. The transcript levels of *ghr-*1 were significantly upregulated in the HF + HT-fed fish with respect to the HF-fed ones, and their levels were lower in the fish fed either diet under restricted feeding conditions. Moreover, *ghr-2* and *igf-1ra* mRNA levels were higher in those animals that received HF + HT diet at a restricted feeding regime compared to HF_RE and HF + HT_ST groups, respectively (Figure 3C).

### 3.3 Gene expression related to myogenesis and osteogenesis

**FIGURE 4:**
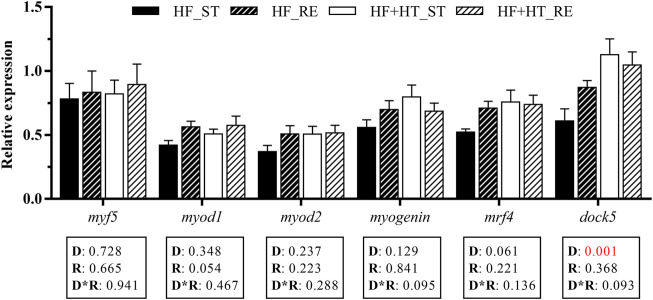
Relative gene expression of myogenic markers in white muscle of gilthead sea bream juveniles fed with a high-fat diet in the absence (HF) or presence (HF + HT) of hydroxytyrosol (0.52 g HT/kg feed), at a standard (ST) (3% biomass/tank) or restricted ration (RE) (40% daily reduction) for 8 weeks. Data are shown as Mean + SEM (*n* = 10). Statistical differences are indicated in 3 components: diet (D), ration (R) and interaction (D*R), using two-way ANOVA (*p* < 0.05, showed in red).

Concerning MRFs, the transcript levels of the dedicator of cytokinesis *dock5* were significantly upregulated in animals fed with the HF + HT diet respect to those fed with the HF diet. On the other hand, the remaining myogenesis-related genes were not altered by either diet composition or feeding regime (Figure 4).

**FIGURE 5:**
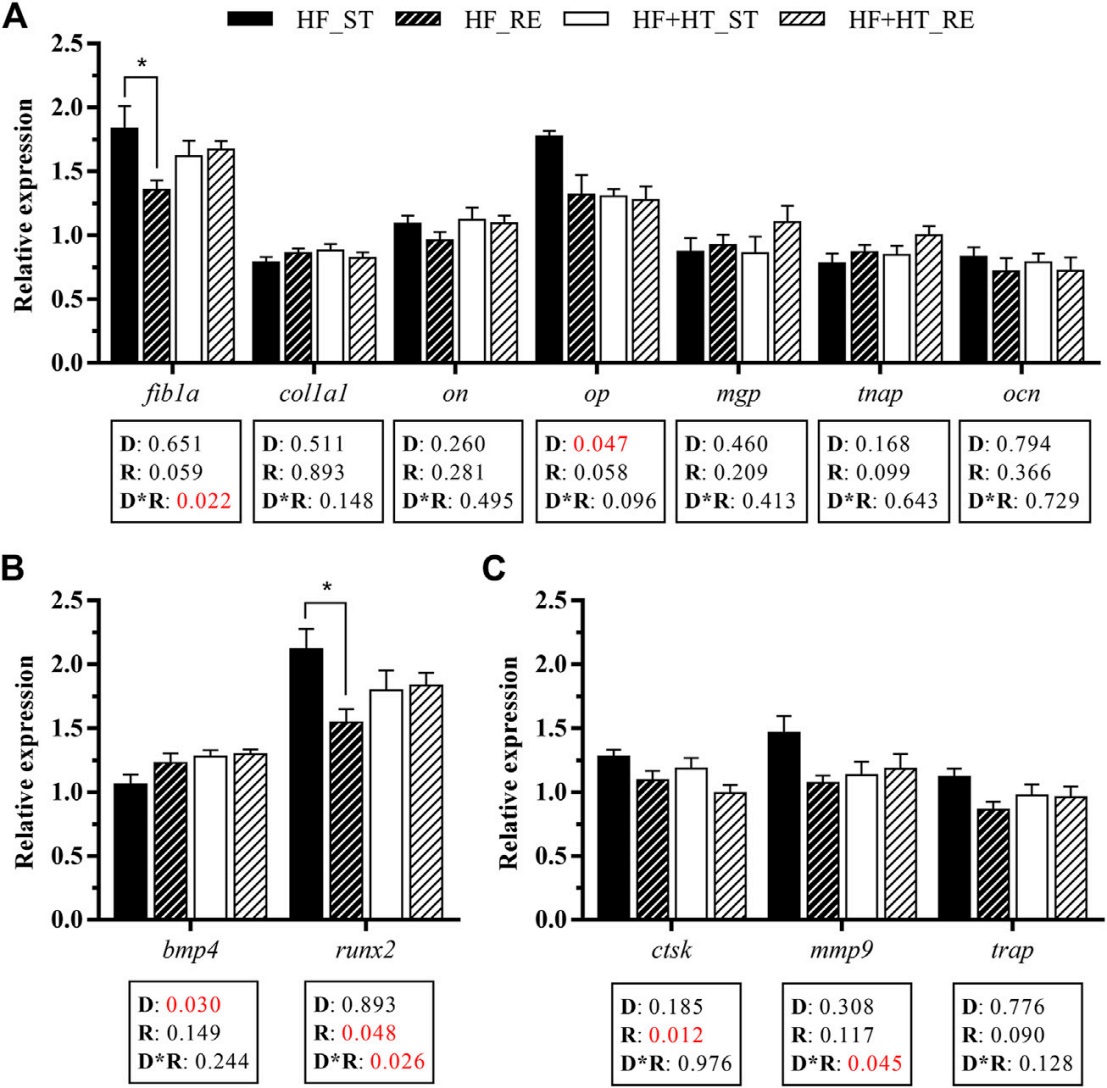
Relative gene expression of **(A)** extracellular matrix components, **(B)** osteogenic and **(C)** osteoclastic markers in bone of gilthead sea bream juveniles fed with a high-fat diet in the absence (HF) or presence (HF + HT) of hydroxytyrosol (0.52 g HT/kg feed), at a standard (ST) (3% biomass/tank) or restricted ration (RE) (40% daily reduction) for 8 weeks. Data are shown as Mean +SEM (*n* = 10). Statistical differences are indicated in 3 components: diet (D), ration (R) and interaction (D*R), using two-way ANOVA (*p* < 0.05, showed in red). When the interaction between the two factors (D*R) was significant, comparisons among groups were analyzed by a Tukey’s post-hoc test and, significant differences among groups are indicated by asterisks (*p* < 0.05).

Gene expression of bone ECM components, and markers of bone turnover in vertebra bone is presented in Figure 5. Regarding the mRNA levels of the matrix components, the interaction between diet and regime conditions significantly affected the gene expression of *fib1a*. Fish fed with the HF diet at the restricted ration showed a downregulated expression of *fib1a* compared to those fish fed with the same diet but at their corresponding standard ration. In addition, the mRNA levels of *op* were modulated by the diet factor, and were significantly higher in fish fed with HF diet respect to the HF + HT-fed group. Besides, the remaining ECM components evaluated did not change in response to any factor (Figure 5A).

Furthermore, the transcript levels of the osteogenic genes *runx2* and the bone morphogenetic protein *bmp4* responded to ration and factors interaction, and only diet factor, respectively. The gene expression of *runx2* was downregulated in the animals fed with the HF diet at the restricted ration in comparison with those fed at the standard one. Moreover, the fish fed the HF + HT diet showed increased mRNA levels of *bmp4* when compared to HF groups (Figure 5B). Concerning the osteoclasts markers, the gene expression of cathepsin k (*ctsk*) and the matrix metalloproteinase *mmp9* was modulated by the ration factor and the interaction between both factors, respectively. The mRNA levels of *ctsk* were lower in the fish fed with both diets under the restricted feeding regime, whereas in the case of *mmp9* the highest transcript levels were observed in the HF-fed fish at standard ration. In contrast, the tartrate‐resistant acid phosphatase (*trap*) mRNA levels remained unaltered (Figure 5C).

### 3.4 Proliferation and differentiation in bone-derived cells

**FIGURE 6:**
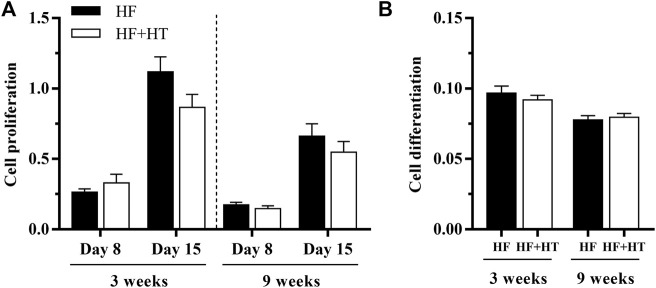
**(A)** Proliferation and **(B)** differentiation of bone-derived cells at days 8 and 15, or 20 respectively, of culture development, extracted from gilthead sea bream juveniles fed with a high-fat diet in the absence (HF) or presence (HF + HT) of hydroxytyrosol (0.52 g HT/kg feed), at a standard ration (3% biomass/tank) for 3 and 9 weeks. Data are shown as Mean + SEM (*n* = 8). No differences were observed between diets, assessed by student-unpaired *t*-tests (*p* < 0.05).

To further investigate the effects of hydroxytyrosol on bone tissue, cell proliferation and differentiation of cultured bone-derived cells obtained from fish fed with the HF and HF + HT diets at a standard ration were analyzed after 3 and 9 weeks of feeding trial. The proliferation data revealed higher rates at day 15 than at day 8, but no differences between groups, regardless of the day of culture development or week of experiment (Figure 6A). In parallel to this, cells coming from fish fed with the HF + HT_ST diet did not present differences in terms of ECM mineralization after 20 days in culture in comparison with cells obtained from animals fed with the HF_ST diet, independently of the experimental week (Figure 6B).

## 4 Discussion

The beneficial properties of hydroxytyrosol or hydroxytyrosol-rich extracts have been widely studied in mammals; however, the effects of this polyphenol in fish functional feeds are less known. In the current study, we evaluated the inclusion of a hydroxytyrosol-rich extract in a high-fat diet on the somatic growth regulation of gilthead sea bream juveniles fed at a standard or reduced ration.

The addition of the hydroxytyrosol-rich extract to the diet did not change BW along the trial, neither any other biometric parameter of the fish after 4 or 8 weeks, regardless of the ration regime. Nonetheless, although IGF-1 plasma levels were relatively low, probably following their seasonal profile (Escobar-Aguirre et al., 2020), dietary hydroxytyrosol was found to significantly affect them, being the levels higher in HF + HT groups, suggesting an anabolic condition (Picha et al., 2008). IGFs are known to be the primary mediators of the growth-promoting effects of GH in fish (Reindl and Sheridan, 2012). In this sense, contrary to GH levels, circulating IGF-1 levels are considered to be one of the most reliable markers of growth performance in a wide variety of fish species (Pérez-Sánchez et al., 2018). A positive correlation between circulating IGF-1 and growth rate was observed in gilthead sea bream after sustained exercise (Vélez et al., 2016) or a recombinant bovine GH injection (Vélez et al., 2018). In fact, the applicability of this parameter as a growth marker is based not only on its relationship to current growth status, but also on its ability to predict future BW gain, as long as feeding and other conditions are unaltered (Pierce et al., 2001; Picha et al., 2008; Izutsu et al., 2022). According to this, it cannot be discarded that an experiment with younger animals (e.g., less than 10 g initial BW) or a prolonged trial could have shown increased growth performance of the animals fed with the HF + HT diet in comparison to the fish fed the same diet but in the absence of hydroxytyrosol. In this context, hydroxytyrosol supplementation (100 mg/kg) on a high-fat diet (15% fat) led to a significant improvement of the BW gain, specific growth rate, FCR and protein efficiency ratios in blunt snout bream (*Megalobrama amblycephala*) juveniles after 10 weeks, together with an alleviation of the excessive lipid accumulation induced by the high-fat diet (Dong et al., 2020). As far as we know, apart from that research work, other *in vivo* studies including hydroxytyrosol *per se* in fish diets have not been reported to date. The fact that the authors include only this polyphenol and not a natural olive extract containing other compounds may explain the high dose used in their study.

Nevertheless, the effects of the dietary inclusion of olive oil products in aquafeeds have been evaluated in several fish species (reviewed by Hazreen-Nita et al., 2022). Likewise, it has been shown that a diet supplemented with an olive oil bioactive extract (rich in polyphenols) promoted growth in a dose-dependent manner in gilthead sea bream juveniles, without affecting feed efficiency parameters (Gisbert et al., 2017). However, the authors proposed that these results could be partially attributed to polyphenols, but also related to the improved intestinal health condition. Indeed, this was also found in rainbow trout (*Onchorynchus mykiss*) after a 6-week feeding trial with an olive waste cake (Hoseinifar et al., 2020). A similar response in terms of growth was also reported in common carp (*Cyprinus carpio*) fed with an olive leaf extract-supplemented diet (Zemheri-Navruz et al., 2020; Sokooti et al., 2021). In contrast, growth performance values, such as BW gain and standard growth rate, or feed utilization were not affected in rainbow trout fed with a diet containing different levels of olive leaf extract for 60 days (Baba et al., 2018). Taken together, these data suggest that a direct link between the polyphenols and somatic growth exists, although differences from various studies may be related to fish species, concentrations of the extracts or the enriched compounds within, the diverse intestinal microbiota and/or experimental conditions (Hazreen-Nita et al., 2022). Overall, the promising effects of the present study should be taken into consideration in the aquaculture sector, since in addition to the positive effects of hydroxytyrosol, gilthead sea bream appears to be more tolerant to high levels of polyphenols in the feed than other fish species (Sicuro et al., 2010).

On the other hand, in our study, fish subjected to 40% feed restriction were unable to maintain the same growth rate as the ones that received the standard ration, regardless of the diet, therefore BW, BL, CF, liver weight, or HSI parameters were significantly lower in that group. However, the feed conversion efficiency in the fish under the restricted regime was improved, indicated by the lower FCR values obtained, which is consistent with previous studies in the same species (Eroldoǧan et al., 2008; Bonaldo et al., 2010). In fact, fish tend to compensate the restricted rations optimizing the digestion process, which allows a more efficient use of the nutrients (Bonaldo et al., 2010).

Parallel to this, the transcriptional regulation of the GH-IGFs-related genes was highly affected by the addition of hydroxytyrosol to the diet, but also by the interaction of diet and feeding regime. The liver showed the most significant differences, compared to the other two tissues studied, given its key role within this endocrine axis and, the benefits of the supplementation with hydroxytyrosol in this tissue, were mostly observed in the fish fed the standard ration; while the restricted conditions, were apparently able to mitigate some of the negative effects induced by the elevated input of lipids received by the animals feeding the high-fat diet. The decrease in the hepatic mRNA levels of most of the genes of the axis (mainly *igf-1*, *igfbp-2* and *igfbp-4*) in the HF + HT dietary groups, but also in the HF_RE group, could be the result of a negative feedback inhibition from circulating IGF-1 after continued signaling by this hormone, as a mechanism to sustain GH/IGF system homeostasis. Also in the liver, the receptors *ghr-1*, *ghr-2* and *igf-1rb* showed the same gene expression pattern as the other members of the axis, indicating a possible GH and IGF-1 desensitization. A similar downregulation in response to high circulating levels of hormones has been previously described in terms of receptor binding in isolated hepatocytes from both, Atlantic salmon (*Salmo salar*) and rainbow trout (Plisetskaya et al., 1993), as well as from lamprey (*Lampetra fluviatilis*) (Leibush and Lappova, 1995). Besides, a downregulation of some hepatic elements of the system (*igf-1, igfbp-1a* and *ifgbp-2)* has been also described when the GH/IGF axis becomes overstimulated after GH administration in gilthead sea bream (Vélez et al., 2019). Interestingly, the contrary effect (i.e., receptors upregulation) has been observed in fish white muscle in response to high insulin levels (Párrizas et al., 1994). This agrees with the highest *ghr-1* mRNA levels found in the HF + HT_ST group in both, white muscle and bone. Apart from this, expression of GH-IGFs-related genes in white muscle was not markedly affected by dietary inclusion of the hydroxytyrosol-rich extract, as reflected by the similar BW observed along the trial between the fish groups fed the two experimental diets, regardless of the ration regime.

Notwithstanding, in white muscle, an interaction between diet composition and feeding ration was found for the paralog *igfbp-5b*, and the highest gene expression values were those in HF_RE and HF + HT_ST groups. In several species, it has been shown that pro-growth stimuli such as amino acids, IGFs or sustained exercise increase the expression of this gene (Salem et al., 2010; Azizi et al., 2016). Hence, this is in accordance with the results of the present study, where the highest IGF-1 plasma levels (at week 8) were found in the same experimental groups with upregulated *igfbp-5b*. In addition, this binding protein is induced during myogenic differentiation and its importance in such a process in teleost fish has been well established (Duan et al., 2010; Garcia de la serrana and Macqueen, 2018). In line with this, the relative expression of the cytoplasmic protein *dock5*, a regulator of the myoblast/myocyte fusion process (Moore et al., 2007; Chen et al., 2020), was also modulated by diet composition. Thus, the increase observed in the HF + HT groups compared to the HF groups in *dock5*, together with the upregulation of *igfbp-5b*, suggest that hydroxytyrosol can have a role in inducing this process of muscle growth. Thus, although histological analysis had been performed to better discern if this could have resulted in an increase in the number (hyperplasia) and/or size (hypertrophy) of multinucleated myofibers in the last term, due to technical problems we could not examine muscle sections as it was designed. In any case, Szychlinska et al. (2019) found a significant increase in muscle hypertrophy in a osteoarthritis rat model fed with an extra virgin olive oil-enriched diet, supporting this hypothesis and the fact that hydroxytyrosol could counteract in muscle the negative changes potentially caused by feeding a high fat diet. Only when applying the 40% feed restriction in the HF + HT group, it seems that some of the beneficial effects of hydroxytyrosol were lost, as it happened for instance with the gene expression of *igfbp-5b* in white muscle; however, this could be explained by the lower absolute quantity of the extract that this experimental group would take in, with respect to the HF + HT_ST fish.

In the bone tissue, the addition of hydroxytyrosol to the diet upregulated the signaling molecule *bmp4* in parallel to decreasing the gene expression of the non-collagenous protein *op*. A positive relationship between *bmp4* expression and ossification has been established in different groups of vertebrates including fish (Chen et al., 2002; Albertson et al., 2005). *bmp4* is expressed in osteoblasts prior to formation of mineralized bone nodules being indicative of new bone formation, although high levels of expression have been also reported in response to elevated temperatures, and linked to increased jaw malformations (Ytteborg et al., 2010; Ma et al., 2016). Indeed, *bmp4* has been shown to play a role in jaw development (Albertson et al., 2005) and more recently, in the determination of intermuscular bone distribution (Su and Dong, 2018). Contrarily, *op* is highly expressed in mineralized tissues but not in soft tissues, and its transcript levels increase during embryonic and larval development (Fonseca et al., 2007; Riera-Heredia et al., 2018); therefore, it could be involved in the control of mineral deposition in fish as well as in mammals. In fact, in MC3T3-E1 murine cells, *op* has been demonstrated to negatively regulate proliferation and differentiation and more interestingly, to do it through interaction with the osteoinductive bone morphogenetic protein, *bmp2* (Huang et al., 2004). Thus overall our data, could suggest that feeding the diet HF + HT induces in fish osteoblasts proliferation while it decreases the advancement towards the maturation step (i.e., mineralization), therefore causing a major bone growth potential.

With regards to feeding regime, the decrease in the mRNA levels of *runx2* and *fib1a*, early markers of osteogenesis in this species (Riera-Heredia et al., 2018), in the animals fed with HF diet at the restricted ration compared to the standard one, suggested a reduced capacity of the cells to proliferate when fish are subjected to this level of feed restriction. However, these genes’ downregulation did not occur in the fish fed with the diet containing hydroxytyrosol, supporting for this phytocompound a potential osteoinductor role in fish, as well as in mammals (García-Martínez et al., 2016). This observation is in agreement with the changes in expression found in bone in the present study concerning the members of the GH-IGF axis namely *igf-1*, *ghr-2* and *igf-1ra*, which presented the highest mRNA levels in the HF + HT group, specially under restricted conditions. Nevertheless, it has to be considered that the positive effect of hydroxytyrosol stimulating osteoblasts proliferation was mostly determined using *in vitro* treatments (García-Martínez et al., 2016) and not all cellular models respond equally, since absence (Hagiwara et al., 2011) or even contrary effects (Anter et al., 2016) have also been reported. Besides, synergistic effects among phenolic compounds present in olives and its derivatives have been suggested to be responsible for osteoblasts proliferation (Chin and Ima-Nirwana, 2016). For this reason, we aimed to evaluate if the *in vivo* administration of the hydroxytyrosol-rich extract could affect osteoblasts development *in vitro*, but neither cell proliferation nor differentiation were significantly changed, although in previous studies it has been demonstrated that the history of the fish could influence the osteogenic process *in vitro* (Balbuena-Pecino et al., 2019).

Finally, in our experimental conditions, the bone-resorbing process by osteoclasts seems not to be modulated at a transcriptional level by the dietary inclusion of hydroxytyrosol-rich extract, since *ctsk* was the only osteoclast gene marker affected by the feeding regime, being its expression reduced in those fish fed with the restricted ration. In mammalian studies, this polyphenol has been reported to inhibit the formation of osteoclasts in a dose dependent manner *in vitro* (Hagiwara et al., 2011) and to prevent bone loss in ovariectomized (osteoporosis model) rats (Puel et al., 2008). Therefore, an *in vitro* system of fish osteoclasts would be key to further evaluate the potential of hydroxytyrosol and other polyphenols in regulating bone turnover, and specifically, resorption.

Summarizing, the bone results may indicate that hydroxytyrosol does not markedly affect bone metabolism in gilthead sea bream, and only somewhat induces growth parameters in the conditions evaluated.

In conclusion, although the fish fed the hydroxytyrosol-rich extract-containing diet (HF + HT) did not show increased somatic growth in comparison to the fish fed the diet without hydroxytyrosol (HF) after 8 weeks, the fish presented elevated IGF-1 plasma levels, as well as increased expression of *igfbp-5b*, *ghr-1* and *dock5* in muscle and *igf-1*, *ghr-1* and *bmp4* in bone together with reduced *op*, suggesting improved growth potential. Therefore, even though further studies should be performed, especially in terms of exploring the specific effects of hydroxytyrosol on muscle and bone structure and remodeling, the present data supports the beneficial use of hydroxytyrosol-rich extracts in functional diets for the optimization of gilthead sea bream aquaculture since the growth capacity of the musculoskeletal system of the fish was improved even if feeding a high-fat diet.

## Data Availability

The data presented in the study are deposited in the CORA repository and can be accessed at: https://doi.org/10.34810/data217.
