# Supplementary material for: Hydroxytyrosol-rich extract from olive juice as an additive in gilthead sea bream juveniles fed a high-fat diet: Regulation of somatic growth
Source: Front Physiol. 2022 Oct 6;13:966175. doi: 10.3389/fphys.2022.966175 (PMC9584614; doi:10.3389/fphys.2022.966175)
Supplement: Supplementary file 1 [file Table1.DOCX]

**Table S1.** Primers used in the Real-Time quantitative PCR analyses.

| **Type** | **Gene** | **Primer sequences (5’–3’)** | | **Ta (ºC)** | **Accession number** |
| --- | --- | --- | --- | --- | --- |
| **Reference** | *rps18* | **F**: GGGTGTTGGCAGACGTTAC  **R**: CTTCTGCCTGTTGAGGAACCA | | 60 | AM490061.1 |
|  | *ef1α* | **F**: CTTCAACGCTCAGGTCATCAT  **R**: GCACAGCGAAACGACCAAGGGGA | | 60 | AF184170 |
|  | *rpl27a* | **F**: AAGAGGAACACAACTCACTGCCCCAC  **R**: GCTTGCCTTTGCCCAGAACTTTGTAG | | 68 | AY188520 |
| **GH-IGFs axis** | *igf-1* | **F**: GAGACCTAGTGGAGGCTGTC  **R**: TCTTGTTGATGGTGAGCTGT | | 60 | EF688016 |
|  | *igf-2* | **F**: TGGGATCGTAGAGGAGTGTTGT  **R**: CTGTAGAGAGGTGGCCGACA | | 60 | AY996778 |
|  | *igfbp-1a* | | **F**: AGTGCGAGTCCTCTCTGGAT  **R**: TCTCTTTAAGGGCACTCGGC | 60 | KM522771 |
|  | *igfbp-2a* | | **F**: CGGGCTGCTGCTGACATACG  **R**: GTCCCGTCGCACCTCATTTG | 60 | AF377998 |
|  | *igfbp-4* | | **F**: TCCACAAACCAGAGAAGCAA  **R**: GGGTATGGGGATTGTGAAGA | 60 | F5T95CD02JMZ9K |
|  | *igfbp-5b* | | **F**: TTTCTCTCTCGGTGTGC  **R**: TCAAGTATCGGCTCCAG | 60 | AM963285 |
|  | *ghr-1* | | **F:** ACCTGTCAGCCACCACATGA  **R**: TCGTGCAGATCTGGGTCGTA | 60 | AF438176 |
|  | *ghr-2* | | **F**: GAGTGAACCCGGCCTGACAG  **R**: GCGGTGGTATCTGATTCATGGT | 60 | AY573601 |
|  | *igf-1ra* | | **F**: AGCATCAAAGACGAACTGG  **R**: CTCCTCGCTGTAGAAGAAGC | 55 | KT156846 |
|  | *igf-1rb* | | **F**: GCTAATGCGAATGTGTTGG  **R**: CGTCCTTTATGCTGCTGATG | 55 | KT156847 |
| **Myogenesis** | *myf5* | | **F**: CTACGAGAGCAGGTGGAGAACT  **R**: TGTCTTATCGCCCAAAGTGTC | 64 | JN034420 |
|  | *myod1* | | **F**: TTTGAGGACCTGGACCC  **R**: CTTCTGCGTGGTGATGGA | 60 | AF478568.1 |
|  | *myod2* | | **F**: CACTACAGCGGGGATTCAGAC  **R**: CGTTTGCTTCTCCTGGACTC | 60 | AF478569 |
|  | *myogenin* | | **F**: CAGAGGCTGCCCAAGGTGGAG  **R**: CAGGTGCTGCCCGAACTGGGCTCG | 68 | EF462191 |
|  | *mrf4* | | **F**: CATCCCACAGCTTTAAAGGCA  **R**: GAGGACGCCGAAGATTCACT | 60 | JN034421 |
|  | *dock5* | | **F**: TCAACAGGCCCAGTAAATCC  **R**: GGGAAGCAGTTCCATCATTC | 60 | SRR278741 isotig14100 |
| **Osteogenesis** | *fib1a* | | **F**: CGGTAATAACTACAGAATCGGTGAG  **R**: CGCATTTGAACTCGCCCTTG | 60 | FG262933 |
|  | *col1a1* | | **F**: GAGATGGCGGTGATGTGGCGGAGTC  **R**: GCCTGGTTTGGCTGGATGAAGAGGG | 68 | DQ324363 |
|  | *on* | | **F**: AGGAGGAGGTCATCGTGGAAGAGCC  **R**: GTGGTGGTTCAGGCAGGGATTCTCA | 68 | AY239014 |
|  | *op* | | **F**: AAAACCCAGGAGATAAACTCAAGACAACCCA  **R**: AGAACCGTGGCAAAGAGCAGAACGAA | 68 | AY651247 |
|  | *mgp* | | **F**: TGTGTAATTTATGTAGTTGTTCTGTGGCATCTCC  **R**: CGGGCGGATAGTGTGAAAAATGGTTAGTG | 68 | AY065652 |
|  | *tnap* | | **F**: CATCGCAACCCTTTTCACAGTCACCCG  **R**: AACAGTGCCCAAACAGTGGTCCCATTAGC | 68 | AY266359 |
|  | *ocn* | | **F**: TCCGCAGTGGTGAGACAGAAG  **R**: CGGTCCGTAGTAGGCCGTGTAG | 60 | AF048703 |
|  | *runx2* | | **F**: ACCCGTCCTACCTGAGTCC  **R**: AGAAGAACCTGGCAATCGTC | 60 | JX232063 |
|  | *bmp4* | | **F**: CACGCCATTGTTCAGACACT  **R**: GCCCTCCACTACCATTTCCT | 60 | FJ436409 |
| **Osteoclastic** | *ctsk* | | **F**: AGCGAGCAGAACCTGGTGGAC  **R**: GCAGAGTTGTAGTTGGGGTCGTAG | 60 | DQ875329 |
|  | *mmp9* | | **F**: ATTCAGAAGGTGGAGGGAGCG  **R**: CATTGGGGACACCACCGAAGA | 60 | AM905938 |
|  | *trap* | | **F**: CTTAATCGTTGCCATCCCTGTG  **R**: CTCCCATCTGCTCTGCTACTTTG | 60 | FM147928 |

F: forward; R: reverse; Ta: annealing temperature; *rps18*: ribosomal protein s18; *ef1α*: elongation factor 1 alpha; *rpl27a*; ribosomal protein L27a; *igf-1*: insulin-like growth factor 1; *igf-2*: insulin-like growth factor 2; *igfbp-1a*: insulin-like growth factor binding protein 1a; *igfbp-2a*: insulin-like growth factor binding protein 2a; *igfbp-4*: insulin-like growth factor binding protein 4; *igfbp-5b*: insulin-like growth factor binding protein 5b; *ghr-1*: growth hormone receptor 1; *ghr-2*: growth hormone receptor 2; *igf-1ra*: insulin-like growth factor 1 receptor a; *igf-1rb*: insulin-like growth factor 1 receptor b; *myf5*: myogenic factor 5; *myod1*: myogenic determination protein 1; *myod2*: myogenic determination protein 2; *myogenin*: myogenin; *mrf4*: myogenic regulatory factor 4; *dock5*: dedicator of cytokinesis 5; *fib1a*: fibronectin subunit 1a; *col1a1*: collagen type 1 alpha‐1; *on*: osteonectin; *op*: osteopontin; *mgp*: matrix gla protein; *tnap*: tissue non‐specific alkaline phosphatase; *ocn*: osteocalcin; *runx2*: runt-related transcription factor 2; *bmp4*: bone morphogenetic protein 4; *ctsk*: cathepsin k; *mmp9*: matrix metalloproteinase 9; *trap*: tartrate‐resistant acid phosphatase.
